# Outcomes after reirradiation of spinal metastasis with stereotactic body radiation therapy (SBRT): a retrospective single institutional study

**DOI:** 10.1093/jrr/rraa058

**Published:** 2020-08-07

**Authors:** Kazuma Sasamura, Ryoko Suzuki, Takuyo Kozuka, Ryoichi Yoshimura, Yasuo Yoshioka, Masahiko Oguchi

**Affiliations:** 1 Radiation Oncology Department, Cancer Institute Hospital of Japanese Foundation for Cancer Research, 3-8-31, Ariake, Koto-Ku, Tokyo, 135-8550, Japan; 2 Department of Radiation Therapeutics and Oncology, Tokyo Medical and Dental University, 1-5-45, Yushima, Bunkyo-Ku, Tokyo, 113-8519, Japan; 3 Department of Radiology, University of Tokyo Hospital, 7-3-1, Hongo, Bunkyo-Ku, Tokyo, 113-8655, Japan

**Keywords:** spinal metastasis, reirradiation, stereotactic body radiation therapy, palliative radiation therapy

## Abstract

This study was aimed at assessing the feasibility and toxicity of using stereotactic body radiation therapy (SBRT) for reirradiation of spinal metastatic tumors. We conducted a retrospective review, from our institutional database, of the data of patients who received reirradiation, with overlap of some prescribed isodose lines to the vertebra from the initial radiation therapy, between 2007 and 2019. We identified 40 patients with spinal metastatic tumors, of whom 2 had 2 metastatic vertebral lesions each, totaling up to 42 target lesions. The median dose to spinal cord at the initial radiation therapy was 30 Gy. SBRT based on the intensity-modulated radiation therapy (IMRT) technique was used for reirradiation to spare the spinal cord. All patients received a prescription dose of 25 Gy in 5 fractions to the planning target volume (PTV). Among the 40 cases who had pain, pain relief was obtained in 24 (60%) after reirradiation. Neurologic improvement was obtained in 8 of 15 cases (53%). The adverse events were evaluated using the Common Terminology Criteria for Adverse Events Version 5.0. Reirradiation was well-tolerated, with only 2 patients experiencing adverse events ≥grade 2 in severity, including 1 patient with grade 3 pain, and another patient with grade 3 spinal fracture. None of the patients developed radiation myelopathy. Our data demonstrated that reirradiation of spinal metastasis using SBRT provided effective pain relief and neurologic improvement, with minimal toxicity.

## INTRODUCTION

Spinal metastases are frequently observed in patients with cancer, with about 5–10% of all patients with cancer reported to develop spinal metastases [1]. Wong *et al*. [2] reported that of 832 autopsies of patients who had died of cancer, 300 (36%) had spinal metastases. Spinal metastases are frequently associated with morbidities, including pain and spinal cord compression with neurologic deficits and pathologic fractures [3]. Of patients with spinal metastases, 10–20% are reported to develop symptomatic spinal cord compression [4]. In cases where surgical intervention is not appropriate, the main treatment method for spinal metastases is conventional external beam radiation therapy (cEBRT) [5]. Local radiation therapy in the treatment of spinal metastases is aimed at palliation of pain, prevention of pathologic fractures, and preventing progression of, or reversal of neurologic deficits [6]. However, sometimes cEBRT is not effective. Several large international trials, with randomization of patients to various low-dose cEBRT regimens, have consistently reported that ~20% of patients will need reirradiation within 3–6 months for failed efficacy of the initial radiation treatment [7]. In addition, sometimes, spinal metastasis develops within the irradiation field of the previous definitive radiation therapy. However, if we adapt cEBRT as the treatment modality for reirradiation in these cases, palliative responses are obtained due to spinal cord tolerance. The Canadian Clinical Trials Group randomized patients with a previous history of conventional radiotherapy for painful bone metastases to 8 Gy/1 fraction or 20 Gy/5 or 8 fractions [8]. The result was an overall pain response rate of ~30% and a complete pain response rate of 8%. To obtain better outcomes, we have adapted stereotactic body radiation therapy (SBRT), a treatment modality based on the intensity-modulated radiation therapy (IMRT) technique for reirradiation of the spine since 2007. IMRT enables us to achieve steep dose gradients around the target, which allows delivery of high radiation doses to the target, while minimizing the dose to the surrounding normal tissues [9]. We hypothesized that SBRT can improve the local control and minimize treatment-related toxicities in patients with a previous history of vertebral irradiation. The purpose of this retrospective study was to evaluate the effectiveness and safety of reirradation of the spine with SBRT. This study was conducted with the approval of the institutional review board of the Cancer Institute Hospital of Japanese Foundation for Cancer Research (approved number: 2019-1126).

## MATERIALS AND METHODS

We administered reirradiation for 45 spinal metastases in 43 patients by SBRT between January 2007 and December 2019. We excluded 3 patients who had been lost to follow-up within 1 month after the end of the reirradiation from the analyses. We conducted a retrospective analysis of the data of the remaining 42 spinal metastases in 40 patients.

In order to offer quick pain relief and/or avoid progression of neurologic symptoms, we attempted to shorten the interval between the day on which the decision to provide reirradiation was made and the day of start of the reirradiation. Basically we obtained not only computed tomography (CT), but also contrast-enhanced thin-slice magnetic resonance imaging (MRI) for the treatment planning, because MRI was necessary to precisely determine the tumor contour. The treatment planning was conducted using the Eclipse treatment planning system (Varian Medical Systems, Palo Alto, CA, USA). For the radiotherapy planning, the patient’s spinal cord, larynx, esophagus, etc., were contoured as the organs at risk (OAR). We contoured the spinal canal as a substitute for the spinal cord. In addition, when an intervertebral foramen near the tumor was not infiltrated by the tumor, the nerve root passing through that foramen was also contoured as an OAR. The gross tumor volume (GTV) was defined as the macroscopic tumor detected on CT and MRI. The clinical target volume (CTV) was defined as the GTV plus a 3-mm margin, and finally the spinal canal was eliminated. The planning target volume (PTV) was defined as the CTV plus a 2-mm margin, excluding the OARs mentioned above. In addition, if the GTV mainly existed within a vertebral body, additional targets called the CTV_sub and PTV_sub were contoured. The CTV_sub was defined as the CTV plus the whole vertebral body which included the GTV. The PTV_sub was defined as the CTV_sub plus a 2-mm margin.

**Fig. 1.**
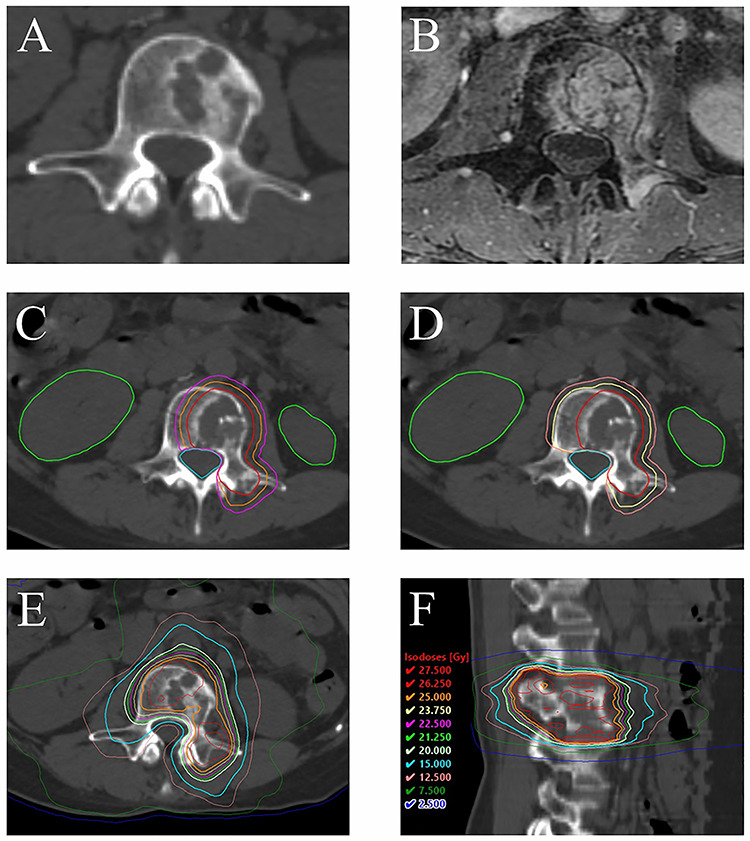
Images, contoured targets and dose distribution in one of the cases: a 46-year-old female descending colon cancer patient with spinal metastases (L3) reirradiated by SBRT. She had undergone palliative radiation therapy for the same spinal metastases at a radiation dose of 30 Gy administered in 10 fractions. The interval between the first radiation therapy and reirradiation was 9.9 months. (**A**) CT showing the tumor before the reirradiation. (**B**) MRI showing the tumor before the reirradiation (contrast-enhanced T1-weighted image with fat suppression). (**C**, **D**) Contouring (red line: GTV; orange line: CTV; purple line: PTV; yellow line: CTV_sub; light pink line: PTV_sub; green line: kidneys; cyan line: spinal cord). (**E**, **F**) dose distribution of the treatment.

For the dose calculation, we used the anisotropic analytical algorithm. SBRT using the IMRT technique was delivered with beams of 6 or 10 MV photons. We attempted to administer the therapy at a total dose of 25 Gy in 5 fractions to 90% of the PTV and more than 20 Gy in 5 fractions to the 95% of the PTV_sub. However, if we could not satisfy these criteria and the dose constraint of the spinal cord, mentioned later, we prioritized the dose constraint of the spinal cord and lowered the dose to the PTV and PTV_sub. For the dose prescription, we considered the tolerance dose of each OAR. The dose constraint of the spinal cord was the Dmax ≤ 20 Gy and D_1 cc_ ≤ 15 Gy. Previously, before introduction of the SBRT approach, we would provide reirradiation for spinal metastases using cEBRT at a prescribed dose of 20 Gy in 10 fractions, and never experienced any severe adverse events, including radiation myelitis. The α/β value for the spinal cord was assumed to be 2 Gy. According to the formula of the linear quadratic model, when the α/β value is 2 Gy, 20 Gy in 10 fractions is biologically equivalent to 15.6 Gy in 5 fractions. Therefore, we considered the constraint of D_1 cc_ of the spinal cord of ≤ 15 Gy as being sufficiently safe to avoid radiation myelitis. The dose constraint of the nerve root was set as the Dmax < 105% of the prescribed dose. The SBRT was delivered using the Clinac 21EX (Varian Medical Systems, Palo Alto, CA, USA) or the TrueBeam (Varian Medical Systems, Palo Alto, CA, USA) linear accelerator. All patients were treated while they lay in the supine position. For fixation, we used Vac-Lok™ cushions. In addition, in cases in which the target tumor was located in the cervical spine or upper thoracic spine, we also used a thermoplastic shell. Prior to July 2007, we used 2D-matching with an on-board imager for verifying the patient position. From October 2007, we have used cone-beam CT for verifying the patient position. Figure 1 is a representative image of a spinal tumor, contoured targets and dose distribution of SBRT.

We evaluated the response in terms of pain relief and amelioration of the neurologic symptoms including paralysis, sensory decline and numbness after reirradiation, by comparing the severity of pain or neurologic symptoms just prior to the start of reirradiation with the best responses obtained after reirradiation. The pain response was evaluated according to the response categories proposed by the International Bone Metastases Consensus Working Party [10]. In addition, in most cases, CT or MRI including the reirradiated region was obtained after the reirradiation about once in a few months, on average, to evaluate the condition of the entire tumor or the therapeutic effect of the treatment. We also evaluated the therapeutic effect of the reirradiation by confirming the change in size of the spinal metastasis on CT or MRI. The local control rate and overall survival rate from the last date of reirradiation were calculated actuarially using the Kaplan–Meyer method. Adverse events were evaluated by the Common Terminology Criteria for Adverse Events, version 5.0. We performed all the statistical analyses using IBM SPSS Statistics version 22.0.

## RESULTS

Patient characteristics are shown in Table 1. The median follow-up time after completion of reirradiation was 9.7 months (range, 1.1–42.8). The characteristics of the first irradiation are shown in Table 2. The median dose at the first irradiation was 30 Gy administered in 10 fractions (range, 9–72 Gy in 3–60 fractions). Of the 42 cases, 2 cases received definitive radiation therapy at the first irradiation, including 1 patient with non-small cell lung cancer with a radiation dose of 45 Gy in 15 fractions. The second patient received definitive radiation therapy for hypopharyngeal cancer at a radiation dose of 72 Gy in 60 fractions (accelerated hyperfractionation of irradiation twice a day). The characteristics of the reirradiation are shown in Table 3. The median interval from the day of CT imaging performed for the treatment planning to day 1 of the first irradiation was 11 days (range 6–21 days). In 9 cases, steroids were used to relieve or prevent neurologic symptoms caused by spinal cord compression.

**Table 1:** Patient characteristics (*n* = 42)

Characteristic	*n* (%)
Age (years), median (range)	66 (43–89)
Gender	
Male	31 (74)
Female	11 (26)
Performance status	
0	5 (12)
1	20 (48)
2	14 (33)
3	3 (7)
Primary cancer site	
Kidney	9 (21)
Lung	8 (19)
Liver	7 (17)
Colon	3 (7)
Thyroid	3 (7)
Others	12 (29)
Spinal region treated	
Cervical	7 (17)
Thoracic	26 (62)
Lumber	9 (21)

**Table 2:** Characteristics of the first irradiation

Characteristic	
Purpose	*n* (%)
Definitive therapy	2 (5)
Palliative therapy	40 (95)
Dose	*n* (%)
30 Gy/10 Fractions	25 (60)
20 Gy/5 Fractions	3 (7)
40 Gy/20 Fractions	3 (7)
Others	11 (26)
	Gy
Dmax of the spinal cord, median (range)	30.0 (7.5–40.0)
BED of the spinal cord^a^, median (range)	75.0 (8.6–95.8)

^a^Biologically equivalent dose, assuming α/β = 2 Gy.

**Table 3:** Characteristics of reirradiation

Characteristic	
Months from the first irradiation	*n* (%)
<6	8 (19)
≥6, <12	14 (33)
≥12	20 (48)
Months from the first irradiation, median (range)	Months
	11.1 (0.6–131.1)
	Gy
D_max_ of the spinal cord, median (range)	19.3 (15.3–25.0)
D_1 cc_ of the spinal cord, median (range)	14.9 (8.9–24.4)
	Gy
BED^a^ of the spinal cord, median (range)	56.7 (38.8–87.8)
Cumulative BED of the spinal cord, median (range)	131.2 (79.5–176.3)

^a^Biologically equivalent dose assuming α/β = 2 Gy.

Of the 42 cases, 40 had pain. After reirradiation, 4 cases (10%) showed complete response, 20 cases (50%) showed partial response, 10 cases (25%) showed indeterminate response, and 6 cases (15%) showed pain aggravation. Of the 42 cases, 15 cases had neurologic symptoms. After reirradiation, improvement of the neurologic symptoms was obtained in 8 cases (53%), no changes were observed in 2 cases (13%), and aggravation of the neurologic symptoms was observed in the remaining 5 cases (33%). Nine cases had neurologic symptoms in the legs. Of these, after reirradiation, 6 patients (67%) could walk, while the remaining 3 patients (33%) could not walk because of aggravation of the neurologic symptoms.

The acute adverse events observed were as follows; radiation dermatitis (grade 1) in 2 cases, muscle weakness of the lower limbs (grade 1) in 1 case, esophagitis (grade 1) in 1 case, malaise (grade 1) in 1 case, anorexia (grade 1) in 1 case, and transient back pain and pain in the extremity (grade 3) in 1 case each. The late adverse events observed were as follows: skin hyperpigmentation (grade 1) in 1 case, telangiectasia (grade 1) in 1 case, and spinal fracture (grade 3) in 1 case. No bone-related adverse events other than spinal fracture were observed. There was no case of radiation myelitis.

**Fig. 2.**
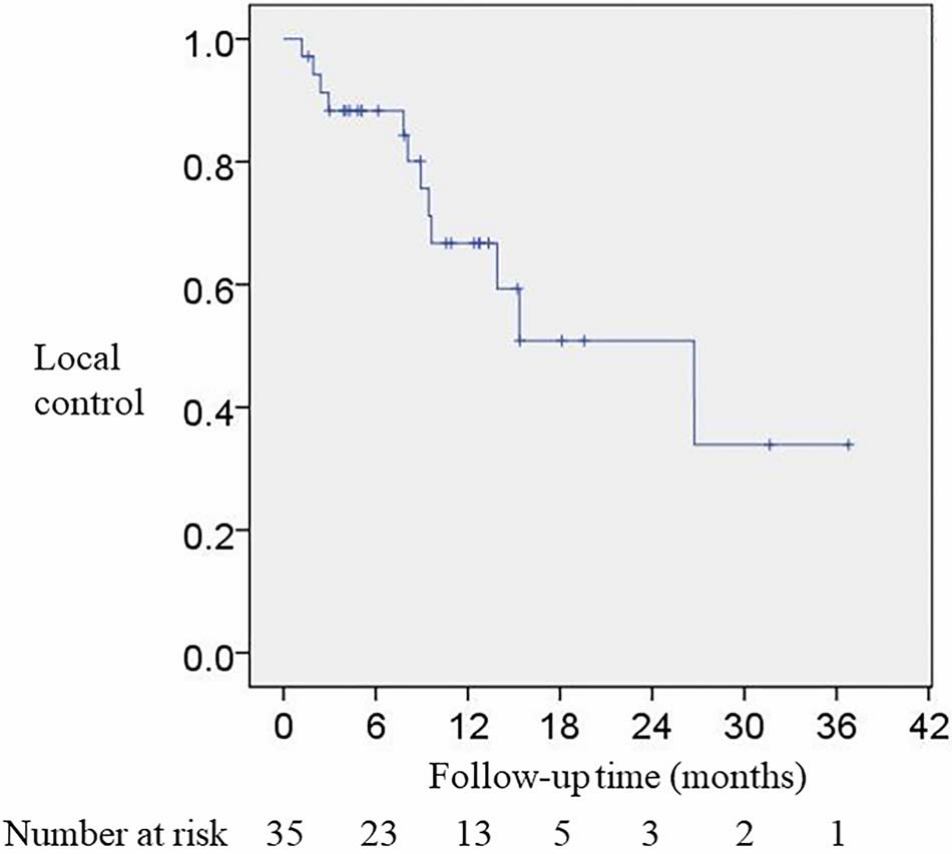
Kaplan–Meier curve of local control after reirradiation.

Of the 42 cases, CT or MRI was performed in 35 cases after reirradiation. As of the last CT/MRI findings, 12 cases (34%) showed local progression, 19 cases (54%) showed no change, and 4 cases (11%) showed reduction in the size of the tumor. The 1- and 2-year local control rates after reirradiation were 67 and 51%, respectively (Fig. 2). The 1- and 2-year overall survival rates were 46 and 15%, respectively.

## DISCUSSION

The American Society for Radiation Oncology (ASTRO) published a guideline for target volume definition in spinal stereotactic radiosurgery in 2012 [11]. Our study had been initiated prior to publication of the ASTRO guideline, therefore, we defined target volume using our own criteria, which were different from those described in the ASTRO guideline. In our study, CTV was defined as the GTV plus a 3-mm margin, whereas in the ASTRO guideline, the definition of CTV differs by the location of the GTV. For example, according to the guideline, if the GTV involves any portion of the vertebral body, the CTV should include the entire vertebral body. In our study, CTV did not include the entire vertebral body in order to reduce the risk of vertebral fracture.

Several studies on reirradiation for spinal metastasis using SBRT have already been published. The reported pain response rates from the previous studies are in the range 65–81% [12–17] and the reported 1-year local control rates were in the range of 66–93% [12–22]. In this study, the pain response rate was 60% and the 1-year local control rate was 67%. Thus, our results appear to be consistent with the results reported from previous studies.

In our study, the neurologic improvement rate after reirradiation was 53%. Thus, reirradiation of spinal metastases by SBRT has the effect of not only reducing the severity of pain, but also of ameliorating neurological symptoms. Milker-Zabel *et al*. [3] analysed 19 cases with spinal tumors who received reirradiation by fractionated conformal radiotherapy or IMRT. They reported a neurologic improvement rate of 42% of patients, which seems compatible with the results of our present study. Boyce-Fappiano *et al*. [12] analysed 237 spine regions that were reirradiated by stereotactic radiosurgery (SRS). The median SRS dose was 16 Gy administered in a single fraction. They reported a neurologic improvement rate after treatment of 82%. Gerszten *et al*. [23] analysed 500 cases with spinal metastasis treated by SRS. They used a mean dose for SRS of 20 Gy, and reported that 84% of their patients with progressive neurologic deficit showed clinical improvement. These latter response rates of 82 and 84% were significantly higher than our results. Although the reason for this difference remains unclear, one may speculate that it might be attributable to the biologically equivalent dose of SRS (41.6–60 Gy assuming α/β = 10 Gy) being higher than that of SBRT in our study (37.5 Gy).

To minimize the risk of adverse events is also important in this treatment. We observed only 1 case of vertebral compression fracture (4.8%) as a late adverse event of grade 3 or more. Previous studies have also reported vertebral compression fractures as adverse effects of this treatment. The reported rate of vertebral compression fractures after reirradiation by SBRT is 0–22% [12–14, 16, 18, 21]. In our study, the rate of vertebral compression fracture was within the range of that reported from previous studies. On the other hand, in some SRS series [12, 14], the reported rates of vertebral compression fractures after reirradiation are in the range 9.3–22%, which seem rather higher than our results obtained using SBRT. This could be derived from the differences in the numbers of fractions used. Faruqi *et al*. [24] indicated that a higher dose per fraction in SBRT is a risk factor for vertebral compression fracture. Therefore, our treatment with 5 fractions might have a favorable effect on the risk of vertebral bone fractures as compared to SRS. An acute pain flare especially during the SBRT was observed in 1 case (4.8%) in our study, which should be considered as an important adverse event. In this case, dexamethasone administration relieved the pain. The incidences of pain flare during SBRT reported from previous studies are in the range 10–68% [25–28]. Thus, the incidence of pain flare incidence in our study was lower than that reported from previous studies. There were no other severe adverse events including radiation myelitis in our study. Overall, the incidence of severe adverse events during or after spinal reirradiation by SBRT was acceptably low.

## CONCLUSIONS

We have reported on the outcomes of reirradiation using SBRT for 42 spinal metastases. Our data showed that reirradiation of spinal metastases by SBRT was effective for pain relief as well as neurologic improvement. In addition, the incidence of severe adverse events was also relatively low.

## CONFLICT OF INTEREST

None declared.
